# Quantitative Susceptibility Mapping Indicates a Disturbed Brain Iron Homeostasis in Neuromyelitis Optica – A Pilot Study

**DOI:** 10.1371/journal.pone.0155027

**Published:** 2016-05-12

**Authors:** Thomas Martin Doring, Vanessa Granado, Fernanda Rueda, Andreas Deistung, Juergen R. Reichenbach, Gustavo Tukamoto, Emerson Leandro Gasparetto, Ferdinand Schweser

**Affiliations:** 1 Diagnostic Imaging, Diagnosticos das Americas DASA, Rio de Janeiro, RJ, Brazil; 2 Departamento de Radiologia, Universidade Federal de Rio de Janeiro UFRJ, Rio de Janeiro, RJ, Brazil; 3 Buffalo Neuroimaging Analysis Center, Department of Neurology, Jacobs School of Medicine and Biomedical Sciences, University at Buffalo, The State University of New York, Buffalo, NY, United States of America; 4 MRI Clinical and Translational Research Center, Jacobs School of Medicine and Biomedical Sciences, University at Buffalo, The State University of New York, Buffalo, NY, United States of America; 5 Medical Physics Group, Institute of Diagnostic and Interventional Radiology, Jena University Hospital, Friedrich Schiller University Jena, Jena, TH, Germany; 6 Michael Stifel Center for Data-driven and Simulation Science Jena, Friedrich Schiller University Jena, Jena, TH, Germany; Medical University of Innsbruck, AUSTRIA

## Abstract

Dysregulation of brain iron homeostasis is a hallmark of many neurodegenerative diseases and can be associated with oxidative stress. The objective of this study was to investigate brain iron in patients with Neuromyelitis Optica (NMO) using quantitative susceptibility mapping (QSM), a quantitative iron-sensitive MRI technique. 12 clinically confirmed NMO patients (6 female and 6 male; age 35.4y±14.2y) and 12 age- and sex-matched healthy controls (7 female and 5 male; age 33.9±11.3y) underwent MRI of the brain at 3 Tesla. Quantitative maps of the effective transverse relaxation rate (R_2_*) and magnetic susceptibility were calculated and a blinded ROI-based group comparison analysis was performed. Normality of the data and differences between patients and controls were tested by Kolmogorov-Smirnov and *t*-test, respectively. Correlation with age was studied using Spearman’s rank correlation and an ANCOVA-like analysis. Magnetic susceptibility values were decreased in the red nucleus (*p*<0.01; *d*>0.95; between -15 and -22 ppb depending on reference region) with a trend toward increasing differences with age. R_2_* revealed significantly decreased relaxation in the optic radiations of five of the 12 patients (p<0.0001; -3.136±0.567 s^-1^). Decreased relaxation in the optic radiation is indicative for demyelination, which is in line with previous findings. Decreased magnetic susceptibility in the red nucleus is indicative for a lower brain iron concentration, a chemical redistribution of iron into less magnetic forms, or both. Further investigations are necessary to elucidate the pathological cause or consequence of this finding.

## Introduction

Neuromyelitis optica (NMO) is a disabling autoimmune astrocytopathy characterized by typically severe and recurrent attacks of optic neuritis and longitudinally extensive myelitis [1,2]. The discovery of the NMO-immunoglobulin G (NMO-IgG) antibody in the serum of patients with this condition and subsequent identification of the target antigen anti-aquaporin-4 (AQP4) [3] suggested that the pathogenesis of NMO differs from that of multiple sclerosis (MS). The presence of these antibodies explains typical central nervous system lesions of NMO, which are located in areas of high AQP expression, being most commonly placed around the ventricles, the hypothalamus, the central canal of the spinal cord, and the optic nerve [4–8].

Advanced neuroimaging techniques have recently become a major research target in NMO due to the hope that they may lead to more specific and sensitive diagnostic criteria for NMO, which allow the unambiguous differentiation of this disease from multiple sclerosis (MS). Diffusion tensor imaging [9,10] and diffusion kurtosis imaging [11] revealed significant alterations of several diffusion parameters [fractional anisotropy (FA), mean diffusivity (MD), axial diffusivity and radial diffusivity] in normal appearing white matter (WM) of patients with NMO when compared to controls, including the pyramidal tract, optic radiations and the corpus callosum (CC). These studies suggest that extensive occult damage in normal appearing white matter (WM) is related to both demyelination and Wallerian degeneration. Changes of diffusion parameters indicate tissue changes in subcortical gray matter (SGM) nuclei such as the thalamus and the putamen [10], indicative of occult damage in these regions. Unfortunately, other advanced imaging techniques, such as MR spectroscopy [12,13] and magnetization-transfer contrast imaging [12,14] show little success in detecting tissue changes in NMO.

A field that remains practically unexplored in NMO is the iron metabolism and the homeostasis of iron in the brain. Oxidative stress is a hallmark of many neurodegenerative diseases, including MS, and is often associated to excess iron [15–17]. Abnormally high iron concentrations have been reported in SGM [18] and lesions of MS patients [19]. For example, using a new iron-sensitive MRI technique called Quantitative Susceptibility Mapping (QSM), Al-Radaideh et al. [20] and Langkammer et al. [21] found increased iron in the caudate nucleus, putamen, globus pallidus, and pulvinar of MS patients. It has been suggested that oxidative stress plays also an important role in the pathogenesis of NMO [22], motivating an investigation of the iron metabolism in the central nervous system of patients with NMO. In a recent study, Chen et al. [23] used a *qualitative* technique that is sensitive toward susceptibility changes, susceptibility-weighted high-pass filtered (HPF) phase imaging, to study SGM iron in NMO. The authors did not find significant differences between patients and normal controls in the SGM (p>0.05). A reason for the negative result of this study may be that HPF phase is a relatively indirect measure of tissue changes that is prone to variations in brain anatomy (e.g. atrophy), post-processing parameters, region-of-interest placement, and head orientation in the MRI scanner [24,25,26].

Contrary to HPF phase, quantitative susceptibility maps (QSM) reflect a local physical tissue property that is independent of experimental parameters and brain anatomy. Magnetic susceptibility of brain tissue is both directly linear to the tissue iron concentration [27] and sensitive to changes in WM integrity [28]. Paramagnetic iron has a positive (hyperintense) and diamagnetic myelin has a negative (hypointense) effect on the numerical voxel values of magnetic susceptibility maps. The effective transverse relaxation rate R_2_* is another MR tissue property that reflects iron and myelin in a localized and quantitative way. R_2_* and QSM are also complementary to each other with iron and myelin both increasing R_2_* [28]. R_2_* and QSM also differ with respect to the spatial scale of structures affecting the measures, with QSM measuring the bulk voxel-average susceptibility and R_2_* reflecting the microscopic heterogeneity of magnetic susceptibility within the voxel.

The purpose of this study was to re-investigate the homeostasis of brain iron in NMO patients using QSM and R_2_* mapping. Our hypothesis was that NMO patients, similar to MS patients, have higher magnetic susceptibility and R_2_* values in SGM nuclei (indicative for iron accumulation) and higher susceptibility and lower R_2_* values in white matter (indicative for demyelination).

## Materials and Methods

### Participants

12 clinically confirmed NMO patients (6 female and 6 male; age 35.4y±14.2y) were selected from the clinical demyelinating disease database at the Federal University of Rio de Janeiro (UFRJ). Inclusion criteria of this retrospective study were (a) patients fulfilled the revised Wingerchuk Criteria of 2006 [1], (b) age was between 14 and 64 years, and (c) no lesions were detected on conventional brain MR images, following the Barkhof criteria for MS [29]. Patients were diagnosed between 2008 and 2010, with mean disease duration of 3 years (ranging from 1–7 years). Five patients had NMO-IgG negative results and 1 had a positive test (using ELISA). Six patients were not tested. The diagnosis was established based on the presence of extensive severe myelitis and/or neuritis, and more than one attack of each in the past. MRI was used to confirm the clinical findings and reject MS, similar to the recommendations for negative or non-tested NMO-IgG patients in the revised NMO diagnostic criteria of 2015 [30]. In addition, we enrolled 12 demographically matched healthy controls (7 female and 5 male; age 33.9±11.3y), free of neurological or psychiatric disorders. All subjects gave written informed consent and the Ethics Committee of the UFRJ approved the study (EC number 169/08).

### MRI

All subjects underwent MRI of the brain at 3 Tesla (Trio Tim, Siemens Medical, Erlangen, Germany) using a 12 channel head coil. In addition to the standard clinical protocol, we acquired an rf-spoiled 3D multi-echo gradient-echo (GRE; T_2_*-weighted imaging) sequence for subsequent conversion of the data to R_2_* and magnetic susceptibility maps. The following acquisition parameters were used: TR = 38 ms; TE_1_ = 4.71 ms (bandwidth 650 Hz/px), TE_2_ = 15 ms (bandwidth 120Hz/px), TE_3_ = 30 ms (bandwidth 120 Hz/px); FOV 180x240x115 mm^3^, matrix size 225x300x96, parallel imaging factor 2, resulting in a voxel size of 0.8x0.8x1.2 mm^3^ with an acquisition time of 9 minutes and 22 seconds. Phase and magnitude images were reconstructed on the scanner and saved separately for each receive channel.

### Reconstruction of susceptibility and R_2_* maps

Multi-channel GRE data were combined according to Ref. [31], resulting phase images were unwrapped and maps of the magnetic field perturbation (the input for QSM) were calculated from the temporal phase evolution using the MCPC-3D-I technique [32]. Background field correction was achieved using V-SHARP [25,33] (radii: 0.8 to 8 mm, TSVD threshold: 0.05) and corrected phase images were converted to susceptibility maps using the HEIDI algorithm [28]. R_2_* maps were calculated from the combined magnitude images using the power method [34] with logarithmic calculus and compensation for macroscopic field gradients [35].

### Image analysis

**Fig 1:**
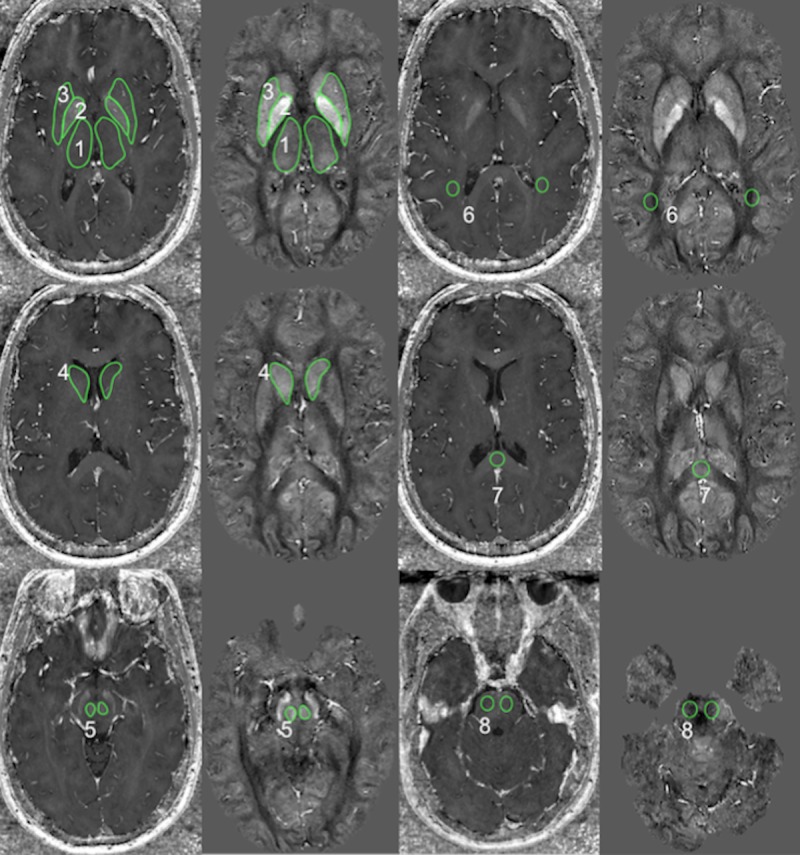
Regions of interest in several cerebral regions. ROIs (green) laid over relaxation rate map and quantitative susceptibility map. Outlined areas included 1: thalamus, 2: globus pallidus, 3: putamen, 4 caudate, 5: red nucleus, 6: optic radiations, 7: corpus callosum, 8: pons. The contrast (black to white) of the relaxation maps ranges from 0 to 80 s^-1^ and of the susceptibility map from -0.096 to 0.186 ppb (relative to the whole brain average).

Two experienced radiologists carried out a blinded region-of-interested (ROI)-based analysis of R_2_* and susceptibility maps within Osirix (version 4.1.2, Pixmeo, Geneva, Switzerland). As illustrated in Fig 1, we defined two-dimensional ROIs by manually outlining SGM regions on the susceptibility maps, because these maps provided the best delineation of the structures. Outlined areas included the thalamus, globus pallidus, putamen, caudate, red nucleus, optic radiations, corpus callosum, and pons. We did not include substantia nigra because of unclear definition of tissue boundaries resulting from the anatomical shape of this region. Subsequently, the ROIs were automatically propagated within OsiriX software to the relaxation maps, which were in the same image space as the susceptibility maps. Fig 1 shows the representative outlines of the manually defined ROIs (green) laid over six slices of the R_2_* relaxation rate map (first and third columns) and quantitative susceptibility map (second and fourth columns). For further statistical analyses we calculated the mean of the susceptibility and relaxation rate values in all ROIs.

QSM provides information about spatial *variations* (differences) of magnetic susceptibility in the brain. Absolute magnetic susceptibilities cannot be extracted. This means that the susceptibility values measured in each subject must be referenced to an internal reference region in each subject before they can be compared to values measured in other subjects. A consequence of this referencing step is that susceptibility changes in the reference region itself have a direct impact on the result of a group comparison study, including both normal inter-subject variations and pathology-related changes. To address this critical limitation, we decided to carry out all susceptibility-related analyses twice with different reference regions: thalamus and caudate, respectively. In both cases we used the average susceptibility value in both hemispheres. Thalamus was chosen because recent studies did not find a difference of susceptibility values in this region between MS patients and controls [21]. Caudate was chosen because it is a highly iron-laden region with relatively low myelin content, minimizing a potential confounding influence of demyelination. With respect to other iron-laden SGM nuclei, the choice of caudate may be regarded arbitrary. We did neither chose the whole brain susceptibility nor WM as a reference region, as was done in previous studies, because NMO is associated with WM damage [9–11], potentially affecting WM susceptibility and leading to false findings. Cerebrospinal fluid (CSF) was not used as a reference region, because i) the choroid plexus has para- (blood) and diamagnetic (calcium) components, ii) segmentation of the ventricles can be difficult in young subjects, and iii) it has previously been shown that CSF introduces additional variation into inter-subject comparisons [26].

### Statistical analysis

All sample distributions were tested for normality using the Kolmogorov-Smirnov test. If sample distributions were normal, Student’s *t*-test was used to determine for each anatomical region if mean values differed between left and right hemispheres (Note: this inter-hemisphere test was not performed in a recent presentation of our preliminary results [36]). If a significant difference was found, further analysis of this region was done for both hemispheres individually; otherwise further analyses used the mean value of left and right hemisphere. Again, when sample distributions were normal, Student’s *t*-test was applied to determine if mean values of controls and patients differed significantly from each other, otherwise the Mann-Whitney U test was used. Significance levels of *p*<0.05 and *p*<0.01 were considered as statistically significant and statistically highly significant, respectively. Ninety-five percent confidence intervals were calculated based on a one-tail t-distribution. We calculated the effect size *d* (Cohen’s d) for each region based on the mean values and standard deviations of each group. Cronbach’s alpha (*α*) was used to assess the inter-rater consistency. Regions with a Cronbach’s alpha below 0.5 were excluded from the analysis.

Knowing that the age-dependence of iron accumulation follows a non-linear trajectory in normal controls [37] we calculated Spearman’s rank correlation coefficient and the two-sided p-value to study the effects of age on susceptibility values. If correlation with age was statistically significant in the control group we investigated if age-dependence of measured values was different between the groups. To this end, we performed an ANCOVA-like analysis that took into account the non-linear aging-trajectory of brain tissue iron [37]. A conventional (linear) analysis of covariances (ANCOVA) cannot directly be applied, because it is known from previous studies that susceptibility varies non-linearly with age. To model the age-dependency of normal controls, we fitted the following exponential function to the mean susceptibility (and correspondingly to R2* values) of controls using a Levenberg-Marquardt algorithm: ${\overset{ˇ}{\chi }}_{\text{control}}(\text{age})=c_{1}∙[1-\exp(-c_{2}∙\text{age})]+c_{0}$, where the (unknown) coefficients *c*_1_ and *c*_2_ describe the age-dependency of susceptibility/R2* and the offset *c*_0_ reflects an (unknown) offset. This function was also used in previous studies to describe the age-dependency of brain iron and susceptibility in white and gray matter [38,39]. For thalamus we used a linear model, because the age-dependency has not yet been established: ${\overset{ˇ}{\chi }}_{\text{control}}(\text{age})=c_{1}∙\text{age}+c_{0}$. To confirm that the respective model properly described the data we tested residuals for normality using the Anderson-Darling test. The resulting (fitted) relationship between age and susceptibility (or R2*) was then used to correct values measured in patients, *χ*_patient_, for normal aging, revealing effects due to disease: $\chi_{\text{patient}}-{\overset{ˇ}{\chi }}_{\text{control}}(\text{age})={\Delta \chi }_{\text{NMO}}(\text{age})$. We applied linear least squares fitting to the resulting values of Δ*χ*_NMO_(age) to describe the age-dependency of disease-related changes. To determine if age-related disease effects were statistically significant we applied an ANCOVA to the values Δ*χ*_NMO_(age) and the residual $\chi_{\text{control}}-{\overset{ˇ}{\chi }}_{\text{control}}(\text{age})$. All calculations were correspondingly performed for R2*.

## Results

**Fig 2:**
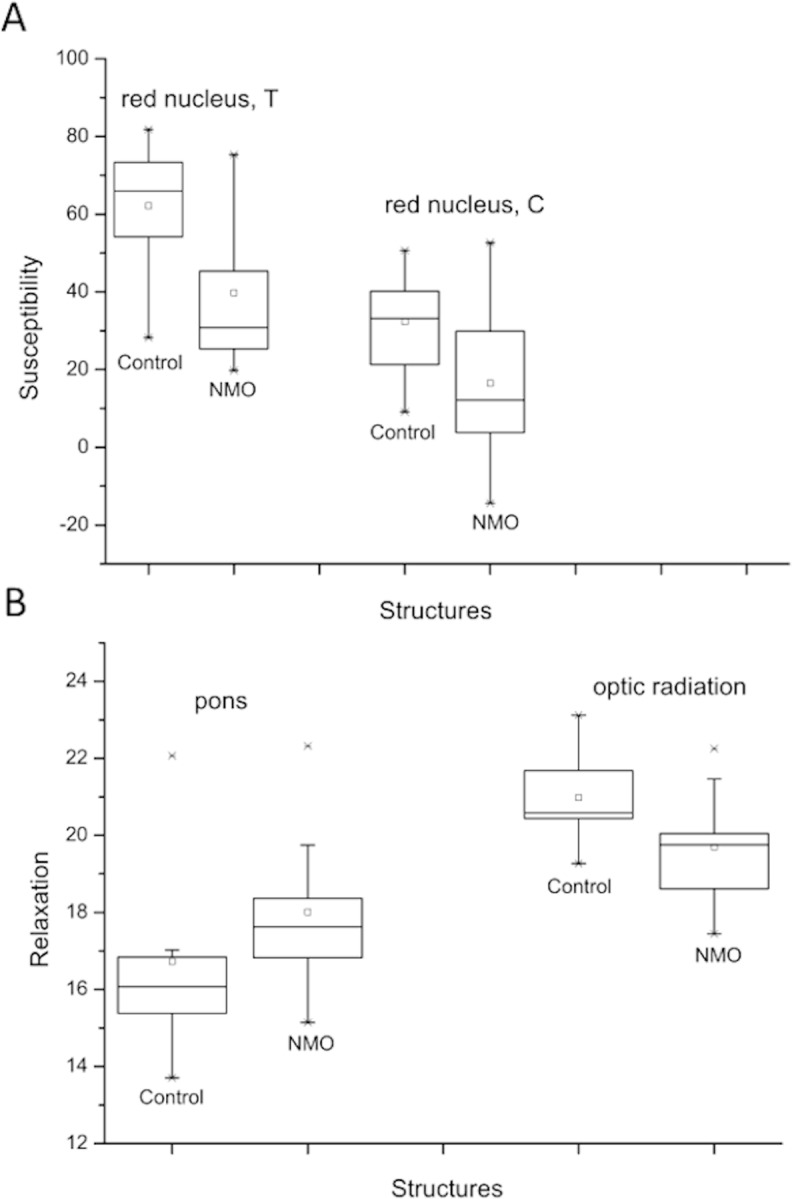
Boxplot. Boxplot of Susceptibilities (A; in ppb) and relaxations (B; in s^-1^) of controls and patients with significant alterations are represented.

**Fig 3:**
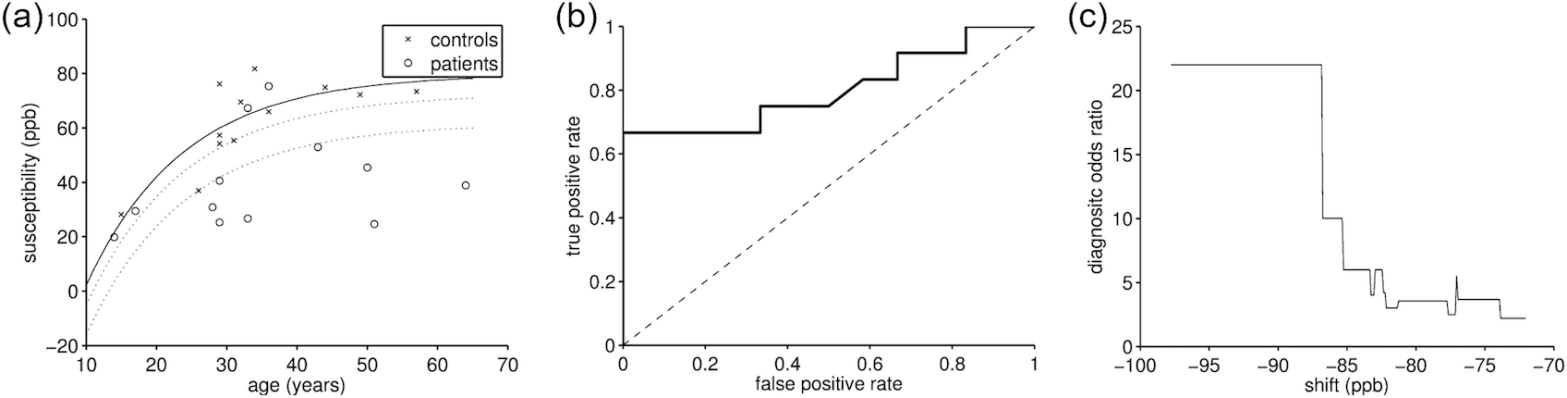
Detailed analysis of susceptibility in the red nucleus. (a) Susceptibility of patients and controls as a function of age. The solid line indicates the fitted normal aging trajectory. The dotted lines represent the area that provided optimal separation of patients and controls. (b) ROC curve of the classification into patients and controls by shifting the aging trajectory in (a, straight line) and considering individuals with lower susceptibility as patients. (c) Diagnostic odds ratio of the separation.

Results are summarized in Tables 1 and 2 and illustrated in Figs 2 and 3. Susceptibility values are reported in parts-per-billion (ppb), R_2_* values are reported in s^-1^.

**Table 1:** Magnetic susceptibilities of controls and patients in different brain regions.

	susceptibility (ppb)			
anatomical region	controls	NMO	*p*	*d*
globus pallidus, T	101.7±13.3 (0.476,0.12)	96.7±16.1 (0.432,0.16)	0.60	0.22
globus pallidus, C	71.9±10.1 (0.127,0.69)	73.5±20.3 (0.098,0.76)	0.88	-0.04
caudate, T	29.8±6.9 (0.816,0.0012**)	23.2±6.7 (0.340,0.28)	0.14	0.67
caudate, C, l	0.0±2.8 (0.156,0.62)	-2.3±3.1 (-0.083,0.79)	0.23	0.50
caudate, C, r	0.0±2.8 (-0.156,0.62)	2.3±3.1 (0.083,0.79)	0.23	-0.50
**red nucleus, T**	62.1±10.4 (0.655,0.021*)	**39.7±11.2** (0.381,0.22)	0.004**	**1.29**
**red nucleus, C**	**32.3±8.5** (0.176,0.58)	**16.5±11.5** (0.267,0.40)	0.024*	**0.96**
putamen, T	43.8±8.6 (0.852,<0.001**)	33.4±11.6 (0.631,0.028*)	0.13	0.69
putamen, C	14.0±5.1 (0.269,0.39)	10.2±10.3 (0.589,0.044*)	0.48	0.33
thalamus, T	0	0	-	-
thalamus, C	-29.8±6.9 (-0.816,0.0012**)	-23.2±6.7 (-0.340,0.28)	0.14	-0.67
corpus callosum, T	5.4±8.3 (0.173,0.59)	9.7±5.7 (0.111,0.73)	0.37	-0.45
corpus callosum, C	-24.4±9.0 (-0.197,0.54)	-13.6±8.5 (-0.216,0.50)	0.072	-0.74

Significantly different and highly significantly different mean values are marked by * and **, respectively. Susceptibility values are stated as mean ± 95% confidence interval (Pearson correlation coefficient *r* with age, p-value of *r*); *p* is the p-value of the group differences; *d* is the effect size. T: Values referenced to bilateral mean of thalamus. C: Values referenced to bilateral mean of caudate nucleus.

**Table 2:** Effective transverse relaxation rates (R_2_*) of controls and patients in different brain regions.

	relaxation rate (1/s)			
Anatomical region	Controls	NMO	*p*	*d*
globus pallidus	36.3±2.8 (0.542,0.068)	37.7±4.8 (0.414,0.18)	0.87	-0.07
caudate	21.1±2.1 (0.563,0.056)	20.6±1.8 (0.611,0.035*)	0.68	0.16
red nucleus	27.2±2.2 (0.775,0.003**)	27.2±2.1 (0.586,0.045*)	0.96	0
putamen	24.8±1.9 (0.740,0.006**)	23.8±2.2 (0.530,0.076)	0.47	0.31
thalamus	19.35±0.67 (0.507,0.092)	19.51±0.81 (-0.586,0.045*)	0.74	-0.08
**optic radiation**	20.99±0.70 (0.796,0.0020**)	**19.70±0.93** (0.102,0.75)	0.025*	**1**
corpus callosum	22.14±0.40 (-0.070,0.83)	21.7±1.2 (0.025,0.94)	0.48	0.32

Significantly different and highly significantly different mean values are marked by * and **, respectively. Relaxation rates are stated as mean ± 95% confidence interval (Pearson correlation coefficient *r* with age, p-value of *r*); *p* is the p-value of the group differences; *d* is the effect size.

We achieved an excellent interrater consistency (*α* ≥ 0.9) in all bilateral basal ganglia nuclei (bilateral globus pallidus, caudate, red nucleus, putamen) and in the left thalamus for both susceptibility and relaxation rate and, only for susceptibility, in right pons and right thalamus. Good interrater consistency (0.9 > *α* ≥ 0.7) was achieved for both measures in CC, and for the relaxation rate in right thalamus and bilateral optic radiations. Consistency was poor (0.6 > *α* ≥ 0.5) for relaxation rate in the right pons and unacceptable (*α* < 0.5; no further analysis) in left pons (both measures) and bilateral optic radiations (susceptibility). Lower consistency in the pons and optic radiations can be attributed to common imaging artifacts in the basal regions of the brain and partial volume and anisotropic susceptibility effects, respectively (see Fig 1).

Mean values in all ROIs followed a normal distribution. Inter-hemispheric differences were found only in the caudate of patients (*p* = 0.03, with caudate reference). The bilateral average values followed a normal distribution in all regions. Independent of the chosen reference region we found (highly) significantly decreased magnetic susceptibility, compared to controls, in the red nucleus of patients (Table 1). The absolute susceptibility differences (and corresponding 95% confidence intervals) were 22.4±15.3 ppb (*d* = 1.29) and 15.7±14.3 ppb (*d* = 0.96) using thalamus and caudate as reference regions, respectively. The relaxation rate was significantly different in the optic radiation, with slower relaxation in patients (-1.3±1.16 s^-1^; *d* = 1.0; Table 2).

Significant correlations of susceptibility with age were found with thalamus reference in putamen (both groups) and in caudate and red nucleus of controls. With caudate reference, susceptibility correlated with age in the thalamus of controls and in the putamen of patients. Similar to these susceptibility-based findings, R2* correlated with age in the red nucleus (both groups), in putamen and optic radiation of controls, and in caudate and thalamus of patients. Fitting of the exponential aging-trajectory to healthy controls resulted in coefficients consistent with the literature for susceptibility of caudate, red nucleus, and putamen with thalamus reference (see S1 and S2 Tables, and S1 Fig). Values were inconsistent for the relaxation rate in red nucleus which may be attributed to instability of the non-linear fitting method. After correction for normal-aging the patient susceptibility values significantly decreased with age (*p*<0.05; all with thalamus reference) in the red nucleus (-0.753±0.337 ppb/year, R^2^ = 41%) and caudate (-0.565±0.208 ppb/year, R^2^ = 38%). Corrected R2* values decreased with age in the optic radiation (0.058±0.032 s^-1^/year, R^2^ = 25%). However, when compared to corrected controls (fitting residuals), neither of these findings reached statistical significance (S2 Table). Visual inspection of the age-corrected scatter plots revealed two clusters of patient R2* values in optic radiation (S2 Fig), one similar to controls and the other with decreased R2*. To study if the clusters had significantly different mean values of R2* and age we defined the clusters by (corrected) R2* > -2s^-1^ and R2* < -2s^-1^, respectively, and performed a two-sample t-tests without assuming equal variances. The difference in R2* values was (3.136±0.567) s^-1^ (95% confidence interval), which was highly significant (p<0.0001). There was no significant difference in age (p = 0.0748).

Since the red nucleus was the only region with significantly different average susceptibility in patients (Table 1) and the region with the strongest age-dependent deviation from controls (S2 Table) we picked this region to study the ability to separate patients and controls based on QSM. To this end we performed a discriminant analysis accounting for the normal aging-related susceptibility increase in healthy individuals. Assuming patients generally have a lower susceptibility in the red nucleus than controls we vertically shifted the normal aging trajectory (parameter *c*_0_) from -100 to +100 ppb (equidistant steps of 0.1ppb) around the optimal parameter determined by fitting to controls (Fig 3A). Considering subjects below the shifted curve as patients and above as normal we calculated a receiver operating characteristic (ROC) curve. The ROC curve is shown in Fig 3B. A 67% sensitivity was achieved at 100% specificity and the area under the curve (AUC) was 0.929 indicating an excellent test method to separate NMO patients from controls. Fig 3C shows the diagnostic odds ratio (DOR) as a function of the shift. The highest odds ratio of 22 was obtained for c_0_ between -98.7 and -86.9ppb, indicated in Fig 3A by dotted lines.

## Discussion

In this pilot study, we investigated brain iron and myelin in NMO patients using two advanced quantitative MR imaging techniques, R_2_* mapping and QSM. Our finding of decreased magnetic susceptibility in the red nucleus suggests a disturbed brain iron homeostasis in patients with NMO.

To the best of our knowledge, decreased magnetic susceptibility has not yet been reported in any neurological disease. Hence, the interpretation of our finding requires a thorough discussion. Tissue magnetic susceptibility can become more diamagnetic (lower numerical values) due to a decrease of the total tissue iron concentration or a redistribution of the present iron into less magnetic forms without a net change of iron abundance. However, ferritin, the major iron storage protein, already has a low magnetic moment relative to other molecules such as transferrin and deoxyheme, making a change of the iron form less probable. Furthermore, a transformation from large amounts of transferrin to ferritin is unlikely because the natural transferrin concentration is relatively low in brain tissues. Another theoretical explanation of decreased magnetic susceptibility could also be a decreased venous vascularization or decreased oxygen extraction fraction (less deoxyhemoglobin) due to, e.g., decreased local metabolic activity. However, it was shown in healthy mice that the contribution of the microvascular network to the voxel susceptibility can generally be neglected [40]. Due to the diamagnetism of myelin, changes of the local myelin concentration represent another confounding factor for the interpretation of our findings. The effect of myelin is opposed to that of paramagnetic iron implying that increased abundance of myelin reduces the voxel’s magnetic susceptibility. Consequently, the observed decrease of magnetic susceptibility in the red nucleus may be interpreted as locally increased myelination. This interpretation would be in line with a recent study by Zhao et al. [10] reporting increased diffusion FA in putamen and thalamus, and reduced MD in the latter. Increased FA and reduced MD are indicative of increased myelination and axonal packing. Although the pathogenesis of NMO is not completely understood, a reasonable explanation for this finding does not exist yet and increased myelin and axonal density in NMO seems contrary to our current understanding that the disease is associated with neurodegeneration [9,11]. Furthermore, related to the reduced susceptibility values found in the present study, the contribution of myelin to the contrast between normal WM and cortical GM on susceptibility maps has been shown to be less than 50 ppb [27]. A susceptibility change of 22.4 ppb or 0.753±0.337 ppb/year (as found in the present study in the red nucleus, referenced to thalamus) seems unlikely to be due to increased myelination alone, although the red nucleus is relatively highly myelinated for a SGM region [41]. Overall, among all these different interpretations, the most probable *(post hoc)* explanation for our finding is a change in brain iron homeostasis leading to a *lower* abundance of iron in the red nucleus. However, an etiological explanation of decreased iron is difficult, because the biochemical role and function of the relatively high iron concentration in the basal ganglia (even in normal subjects) is still unknown [42]. Although our age-dependent results did not reach statistical significance, they indicate that iron in the red nucleus (and caudate) is not increasing to the same degree with age as it does in controls (S2 Table). This trend may indicate a reduced iron uptake below the normal level in NMO, but requires further research using larger groups.

A very recent study found decreased serum iron and increased serum ferritin in NMO patients compared to controls [43]. While the decrease of iron in serum is in line with our findings of decreased iron in the red nucleus, the relation between serum iron and brain iron remains unclear, because iron exchange between the central nervous system and circulation is tightly regulated in humans and serum brain iron usually does not correlate with serum iron [44].

Decreased R_2_* can be explained by demyelination (see Methods) [45]. Hence, decreased R_2_* in the optic radiation of patients is in line with previous studies showing WM damage in these regions [9,11]. Our finding of significantly decreased R_2_* in only five out of twelve patients indicates that not all patients are affected by this pathology. Changes of R_2_* can also reflect increased free water content, a change of the iron concentration, a redistribution of iron into other compartments, or a change of the chemical form of iron. The lack of a significant reduction of R_2_* in the red nucleus is in line with the common assumption that QSM is more sensitive to iron-related tissue changes than R_2_* [21].

With a relatively large effect size (*d* = 1.29; thalamus reference), the change of red nucleus susceptibility may have *practical* significance. Our preliminary ROC analysis (Fig 3) demonstrated an excellent discrimination between patients and controls based on susceptibility in the red nucleus. The finding of decreased susceptibility only in the red nucleus and no significant changes in other regions requires special attention. Application of the QSM technique in patients with MS in two recent studies revealed consistently increased magnetic susceptibility in caudate nucleus, putamen, globus pallidus, and pulvinar [20,21]. While these studies did not investigate susceptibility in the red nucleus, the lack of significant differences in other nuclei in our study may reflect different pathological mechanisms in MS and NMO and bears the potential of a QSM-based differentiation between the two diseases. Reported effect sizes of susceptibility differences between MS and controls were between 0.75 (globus pallidus) and 0.97 (caudate) (calculated using G*Power [46] from mean and standard deviations in [20]). To detect these effects with 80% power (at 5% significance level; one tailed; Wilcoxon-Mann-Whitney test) would require group sizes of 24 and 15 subjects, respectively. Future, studies with larger cohorts will investigate the biomarker potential of QSM involving both MS and MNO patients.

Our pilot study has some limitations. One is that half of the patients were not tested for NMO-IgG, and the other half used an immune assay with lower sensitivity than the serological immune tests now applied (30). This limitation was balanced by the fact that we have had rigorous clinical and MRI criteria diagnosis for NMO. Furthermore, our study had limited statistical power due to a cross-sectional study design and the relatively small and heterogeneous patient cohort when considering clinical data and treatment. This is a general issue in many NMO-related studies due to the relative rarity of the disease. A *post hoc* power analysis showed that the mean power of our study to detect effect sizes of 0.2, 0.5 and 0.8 (with 12 subjects per group at a 5% significance level; two tailed) was only 7%, 21% and 28%, respectively. Hence, the risk that small effects were missed (type II errors) was high. To avoid further increasing the risk of type II errors we did not correct our p-values for multiple comparisons (Bonferroni/Benjamini-Hochberg). Considering that 19 group comparisons were performed (Tables 1 and 2) the Bonferroni corrected significance level would be p<0.0026. However, even with this corrected level red nucleus (thalamus reference) would have still been identified as significantly different between patients and controls.

An *a priori* power analysis (one-tailed; Wilcoxon-Mann-Whitney t-test; allocation ratio one) shows that assuming an effect size of *d* = 1.29 (thalamus reference; Table 1) of susceptibility changes in the red nucleus, a replication of our study would require 14 subjects per group. A validation of our finding in the red nucleus with a 0.1% significance level and 99.6% power would require *N* = 51 subjects per group. However, considering an increasing difference between red nucleus susceptibility in patients and controls, a higher effect size may be achieved in groups with higher average age than in the current cohort, potentially allowing the reproduction of our results in smaller cohorts.

Another limitation of our study is that R_2_* was calculated from only three gradient echoes. A higher number of gradient echoes generally results in R_2_* values with higher precision. The relatively low number of echoes acquired in this study could explain why R_2_* presented as a relatively insensitive measure. In addition, sensitivity of both R_2_* and QSM would benefit from higher field strength. A limitation of the manual definition of the ROIs is that only a limited portion of the brain can be investigated and only average quantities are obtained for the defined regions. Susceptibility variations within the ROIs cannot be analyzed beyond statistical measures. A voxel-based analysis (VBA) would overcome these limitations but requires complex adjustments of current VBA algorithms, which have traditionally been developed for T1w images and cannot handle susceptibility maps with small (negative) image intensity values.

In conclusion, this study presents the first imaging finding that suggests a disturbed iron homeostasis in patients with NMO. The finding of significantly decreased iron concentration in the red nucleus of NMO patients (with a relatively high effect size) promises brain iron imaging could contribute to a better understanding of NMO physiopathology. However, further investigations in larger patient cohorts as well as histochemical analyses are required to confirm the feasibility of QSM as a new imaging biomarker in NMO, understand the pathophysiological cause of the iron changes, and whether QSM may ultimately improve clinical diagnosis of NMO.

## Supporting Information

S1 Fig Scatter plots of susceptibility (top and middle rows) and R2* (bottom row) over age for all control regions in which a significant correlation with age was found. The straight lines represent the fitted aging trajectories (fitting parameters listed in S2 Table).
(TIF)

S2 Fig Susceptibility (left) and R2* (right) values corrected for normal aging using the fitting coefficients listed in S2 Table. The dashed line represents a linear fit to the control group indicating that the correction for normal aging was successful. The straight line represents a linear fit to the corrected patient values. Corresponding fitting coefficients are listed in S3 Table. The dashed horizontal line in the right-most panel indicates the threshold used to define the two clusters of optic radiation R_2_* values.
(TIF)

S1 Table Coefficients resulting from fitting the normal aging trajectory to controls. The coefficients c_0_, c_1,_ and c_2_ are explained in the test. For thalamus, a linear model was fitted instead of the exponential model used for all other regions. Values are stated as regression coefficient ± 95% confidence interval. Values significantly different from zero are indicated by *. R^2^ is the goodness-of-fit.
(DOCX)

S2 Table Comparison of aging-coefficients from literature. Coefficients are stated as regression value ± 95% confidence interval. Coefficient c_3_ not reported for susceptibility, because it cannot be compared between studies due to different reference regions. Vales were taken from (a) Ref. [44] (male and female, all data), (b) Ref. [46], and (c) Ref. [45].
(DOCX)

S3 Table Analysis of differences in age-dependency of susceptibility and R2* values between the two groups in different brain regions. Listed are the slope and offset of the linear fits applied to the normal-aging corrected patient values Δ*χ*_NMO_(age) (and correspondingly for R2*). Slope and offset of linear fits are stated as regression value ± standard fitting error. Slopes significantly different from zero (*p*<0.05) are marked by *. R^2^ denotes the goodness-of-fit. The p-values relate to a comparison of normal-aging corrected control and patient values (see text for details).
(DOCX)
