# Supplementary figures and images for: Quantitative Susceptibility Mapping Indicates a Disturbed Brain Iron Homeostasis in Neuromyelitis Optica – A Pilot Study
Source: PLoS One. 2016 May 12;11(5):e0155027. doi: 10.1371/journal.pone.0155027 (PMC4865155; doi:10.1371/journal.pone.0155027)

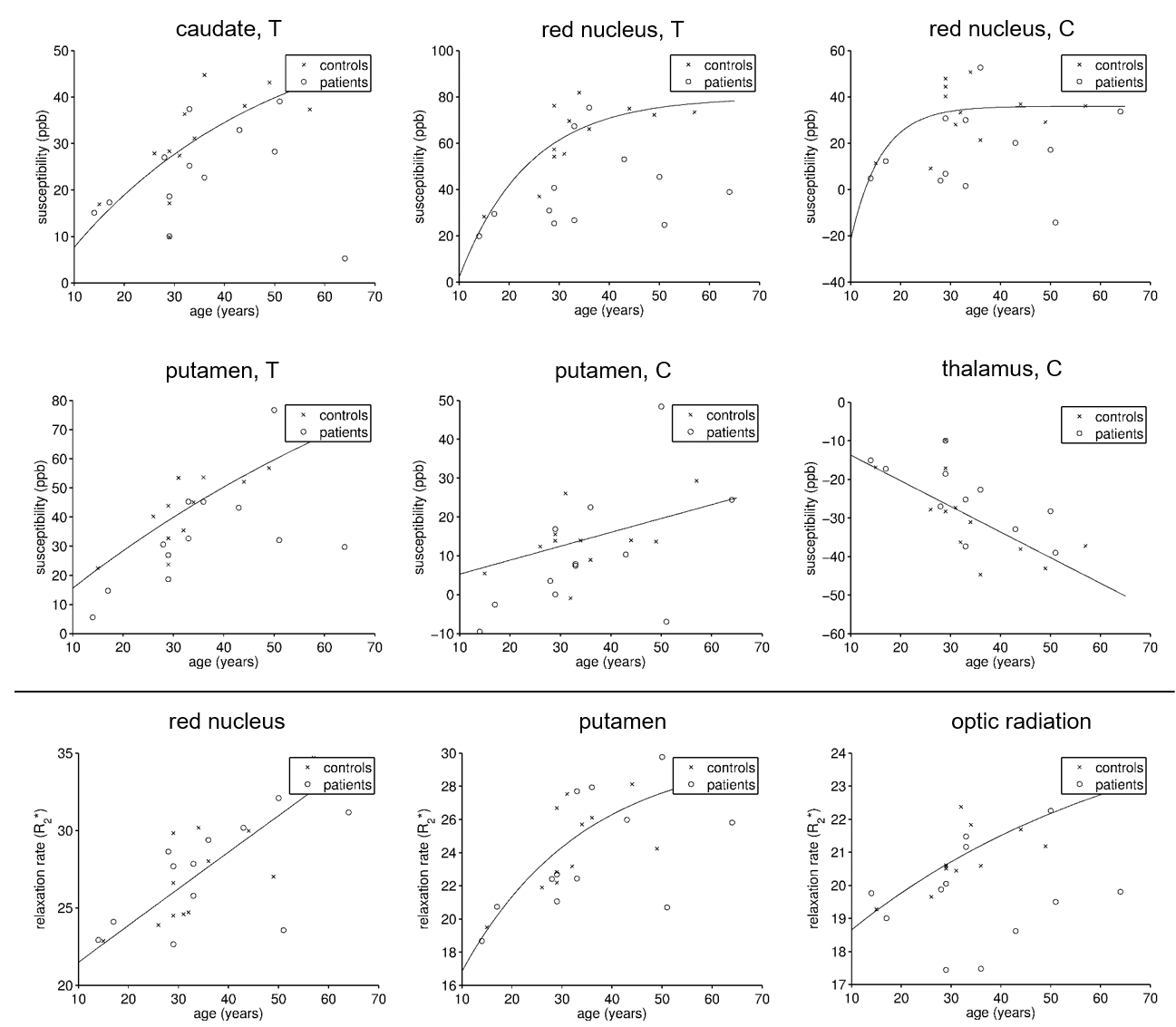

Supplement: S1 Fig — The straight lines represent the fitted aging trajectories (fitting parameters listed in S2 Table). (TIF) [file pone.0155027.s001.tif]

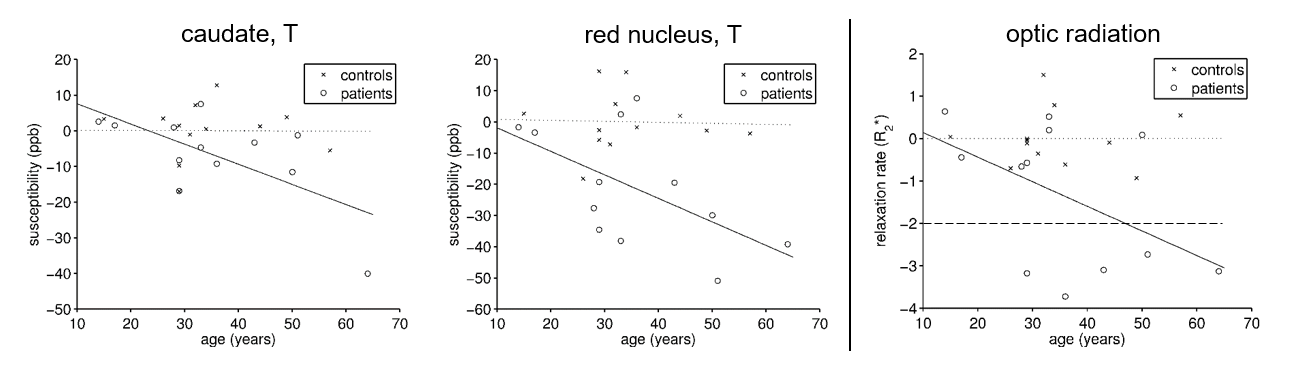

Supplement: S2 Fig — The dashed line represents a linear fit to the control group indicating that the correction for normal aging was successful. The straight line represents a linear fit to the corrected patient values. Corresponding fitting coefficients are listed in S3 Table. The dashed horizontal line in the right-most panel indicates the threshold used to define the two clusters of optic radiation R2* values. (TIF) [file pone.0155027.s002.tif]
