# Supplementary material for: Quantitative Susceptibility Mapping Indicates a Disturbed Brain Iron Homeostasis in Neuromyelitis Optica – A Pilot Study
Source: PLoS One. 2016 May 12;11(5):e0155027. doi: 10.1371/journal.pone.0155027 (PMC4865155; doi:10.1371/journal.pone.0155027)
Supplement: S1 Table — The coefficients c0, c1, and c2 are explained in the test. For thalamus, a linear model was fitted instead of the exponential model used for all other regions. Values are stated as regression coefficient ± 95% confidence interval. Values significantly different from zero are indicated by *. R2 is the goodness-of-fit. (DOCX) [file pone.0155027.s003.docx]

| **anatomical region** | **c_0_** | | | **c_1_** | | | **c_2_** | | | ***R*^2^** |
| --- | --- | --- | --- | --- | --- | --- | --- | --- | --- | --- |
| **susceptibility** | **(ppb)** | | | **(ppb)** | | | **(1/year)** | | |  |
| caudate, T | -6.74 | ± | 74.47 | 65.40 | ± | 70.40 | 0.02489 | ± | 0.10528 | 0.49 |
| red nucleus, T | -79.55 | ± | 214.79 | 159.23 | ± | 196.81 | 0.072005 | ± | 0.0895 | 0.66 |
| putamen, T | 1.30 | ± | 55.17 | 142.70 | ± | 567.38 | 0.010498 | ± | 0.0659 | 0.71 |
|  | **(ppb)** | | | **(ppb/year)** | | |  |  |  |  |
| thalamus, C | -7.10 | ± | 18.35 | **-0.66** | **±** | **0.51*** | (linear model) | | | 0.46 |
| **R2*** | **(1/s)** | | | **(1/s)** | | | **(1/year)** | | |  |
| red nucleus | **19.09** | **±** | **5.17*** | **596.22** | **±** | **5.4e-5*** | **0.000402** | **±** | **0.000248*** | 0.42 |
| putamen | 10.08 | ± | 23.78 | **20.08** | **±** | **16.53*** | 0.0411 | ± | 0.0868 | 0.59 |
| optic radiation | **17.33** | **±** | **5.70*** | 8.45 | ± | 17.22 | 0.01698 | ± | 0.0797 | 0.62 |
