# Supplementary material for: Quantitative Susceptibility Mapping Indicates a Disturbed Brain Iron Homeostasis in Neuromyelitis Optica – A Pilot Study
Source: PLoS One. 2016 May 12;11(5):e0155027. doi: 10.1371/journal.pone.0155027 (PMC4865155; doi:10.1371/journal.pone.0155027)
Supplement: S2 Table — Coefficients are stated as regression value ± 95% confidence interval. Coefficient c3 not reported for susceptibility, because it cannot be compared between studies due to different reference regions. Vales were taken from (a) Ref. [44] (male and female, all data), (b) Ref. [46], and (c) Ref. [45]. (DOCX) [file pone.0155027.s004.docx]

| **anatomical region** | **c_1_** | | | **c_2_** | | | **c_3_** |
| --- | --- | --- | --- | --- | --- | --- | --- |
| **susceptibility** | **(ppb)** | | | **(1/year)** | | |  |
| caudate^a^ | 60 | ± | 10 | 0.05 | ± | 0.02 | - |
| red nucleus^a^ | 120 | ± | 30 | 0.06 | ± | 0.03 | - |
| putamen^a^ | 200 | ± | 140 | 0.01 | ± | 0.01 | - |
| **R2*** | **(1/s)** | | | **(1/year)** | | | **(1/s)** |
| caudate^a^ | 18 | ± | 3 | 0.02 | ± | 0.02 | 12±4 |
| caudate^b^ | 2.13 | | | 0.05 | | | 17.3 |
| red nucleus^a^ | 28 | ± | 10 | 0.06 | ± | 0.03 | 8±11 |
| putamen^a^ | 49 | ± | 74 | 0.02 | ± | 0.02 | 12±4 |
| putamen^b^ | 7.26 | | | -0.03 | | | 18.3 |
| **iron** |  | | | **(1/year)** | | |  |
| caudate^c^ |  | | | 0.05 | | |  |
| putamen^c^ |  | | | 0.04 | | |  |
