# Supplementary material for: Quantitative Susceptibility Mapping Indicates a Disturbed Brain Iron Homeostasis in Neuromyelitis Optica – A Pilot Study
Source: PLoS One. 2016 May 12;11(5):e0155027. doi: 10.1371/journal.pone.0155027 (PMC4865155; doi:10.1371/journal.pone.0155027)
Supplement: S3 Table — Listed are the slope and offset of the linear fits applied to the normal-aging corrected patient values ΔχNMO(age) (and correspondingly for R2*). Slope and offset of linear fits are stated as regression value ± standard fitting error. Slopes significantly different from zero (p<0.05) are marked by *. R2 denotes the goodness-of-fit. The p-values relate to a comparison of normal-aging corrected control and patient values (see text for details). (DOCX) [file pone.0155027.s005.docx]

|  |  | | |  | | |  | **ANCOVA** | |
| --- | --- | --- | --- | --- | --- | --- | --- | --- | --- |
|  | **slope** | | | **offset** | | | ***R*^2^** | ***p, slope*** | ***p, offset*** |
| **anatomical region, susceptibility** | **(ppb/year)** | | | **(ppb)** | | |  |  |  |
| caudate, T | **-0.565** | **±** | **0.208*** | 13.16 | ± | 7.93 | 0.38 | 0.09 | 0.27 |
| red nucleus, T | **-0.753** | **±** | **0.337*** | 5.57 | ± | 12.87 | 0.41 | 0.13 | 0.79 |
| putamen, T | -0.283 | ± | 0.313 | -1.32 | ± | 11.94 | 0.03 | 0.49 | 0.93 |
| putamen, C | 0.278 | ± | 0.293 | -14.14 | ± | 11.10 | 0.07 | 0.47 | 0.32 |
| thalamus, C | 0.571 | ± | 0.229 | -12.83 | ± | 8.72 | 0.38 | 0.10 | 0.31 |
| **anatomical region, R2*** | **(s^-1^/year)** | | | **(1/s)** | | |  |  |  |
| caudate | -0.053 | ± | 0.042 | 1.12 | ± | 1.59 | 0.14 | 0.49 | 0.69 |
| red nucleus | -0.096 | ± | 0.058 | 3.05 | ± | 2.20 | 0.22 | 0.28 | 0.35 |
| putamen | -0.057 | ± | 0.058 | 1.08 | ± | 2.22 | 0.09 | 0.49 | 0.72 |
| optic radiation | **-0.058** | **±** | **0.032*** | 0.72 | ± | 1.22 | 0.25 | 0.17 | 0.99 |
